# Effect of atorvastatin gel in non-surgical treatment of peri-implant mucositis: A randomized controlled clinical trial

**DOI:** 10.34172/japid.2024.017

**Published:** 2024-09-11

**Authors:** Azin Khorramdel, Katayoun Mogharrab Alile, Yousef Kananizadeh, Seyed Amin Mousavi, Fatima Molavi

**Affiliations:** ^1^Department of Periodontics, Faculty of Dentistry, Tabriz University of Medical Sciences, Tabriz, Iran; ^2^Dentist, Faculty of Dentistry, Tabriz Medical Sciences, Islamic Azad University, Tabriz, Iran; ^3^Department of Oral and Maxillofacial Surgery, Faculty of Dentistry, Tabriz Medical Sciences, Islamic Azad University, Tabriz, Iran; ^4^Department of Prosthodontics, Faculty of Dentistry, Tabriz University of Medical Sciences, Tabriz, Iran; ^5^Department of Pharmaceutics, Faculty of Pharmacy, Tabriz University of Medical Sciences, Tabriz, Iran

**Keywords:** Atorvastatin, Clinical trial, Dental implants, Mucositis

## Abstract

**Background.:**

Peri-implant diseases, such as peri-implant mucositis and peri-implantitis, are inflammatory conditions caused by biofilms that can lead to the loss of surrounding soft tissues and bone. The most effective treatment involves non-surgical mechanical debridement to remove plaque, but other treatment modalities have shown limited success. This study investigated the anti-inflammatory and immunomodulatory effects of atorvastatin (ATV) gel as an additional treatment for peri-implant mucositis.

**Methods.:**

In this double-masked, randomized clinical trial, 49 patients with peri-implant mucositis were randomly divided into two treatment groups: mechanical debridement (MD)+placebo or MD+ATV gel. At baseline, 1 month, and 3 months after the intervention, periodontal parameters, including probing depth (PD), bleeding on probing (BOP), clinical attachment level (CAL), and pain on probing (POP), were measured. Data were analyzed using independent t-test and paired t-test.

**Results.:**

Statistically significant improvements in CAL and POP were observed from baseline to each time point throughout the study period (*P*≤0.001). PD and BOP were statistically significant 1 month and 3 months after the intervention, respectively (*P*<0.05).

**Conclusion.:**

The clinical parameters associated with peri-implant mucosal inflammation further improved when ATV gel was used with MD.

## Introduction

 Dental implant therapy is a popular method for replacing missing teeth; however, it can lead to technical and biological complications known as peri-implant diseases.^1^ These biofilm-induced inflammations affect soft and hard bone tissues around osseointegrated implants. There are two categories of peri-implant diseases: peri-implant mucositis and peri-implantitis.^2^ Peri-implant mucositis is a reversible inflammation of the mucosa around the implant,^3^ while peri-implantitis involves progressive bone loss.^4^ The prevalence of peri-implant mucositis ranges from 23.9% to 88%, and peri-implantitis varies from 8.9% to 45%.^5,6^ Although plaque accumulation is the main cause,^7^ other risk factors include smoking, history of periodontitis, lack of regular periodontal maintenance, diabetes, implant design or surface characteristics, and excess cement.^8,9^

 The standard approach for treating peri-implant mucositis is non-surgical treatment, which involves reinforcing oral hygiene practices, including professional and patient-administered plaque control techniques to mechanically remove microbial plaques from the implant surfaces. Studies have investigated adjunctive or alternative methods and non-surgical mechanical debridement for treating peri-implant mucositis.^10^ These methods include antimicrobial photodynamic therapy, antiseptics, topical or systemic antibiotics, abrasive devices, laser therapy, home care mouthwashes, and probiotics.^11-14^ However, it should be emphasized that regardless of the treatment used, adequate plaque control is important for the complete resolution of the condition.^15^

 Statins, commonly prescribed for lower lipid levels to prevent cardiovascular events, have shown potential for treating periodontal diseases. Studies have demonstrated that statins can reduce tooth mobility, tooth loss, and bone resorption in patients with chronic periodontitis. In addition to their lipid-lowering effects, statins possess anti-inflammatory, immunomodulatory, antioxidant, antithrombotic, and endothelium-stabilizing properties. They can also promote angiogenesis and stimulate bone formation.^16,17^ Recent studies have shown that patients receiving statin treatment for chronic periodontitis have fewer pathological periodontal pockets than those not receiving such medication. Atorvastatin (ATV), in particular, demonstrates inhibitory effects on inflammatory cells and matrix metalloproteinases that play a crucial role in degrading connective tissue in periodontal diseases.^16^

 Akram et al.^18^ found that 1.2% ATV gel applied locally improved clinical and radiographic parameters significantly. ATV was more effective than other statins, such as simvastatin, pravastatin, lovastatin, and fluvastatin, in lowering low-density lipoprotein. According to this study, ATV may be more effective than simvastatin in promoting bone regeneration in periodontal defects, reducing probing depth (PD) and clinical attachment level (CAL), and exerting anti-inflammatory effects.^17,19^ Although none of the recent studies demonstrated the effectiveness of ATV gel once it is used locally to treat peri-implant mucositis, the present study was undertaken to evaluate the effectivity of 1.2% ATV gel in addition to mechanical debridement for the treatment of peri-implant mucositis. This study was necessary as statins are very effective in anti-inflammatory effects, and a limited number of studies have been conducted in this field.

## Methods

###  Study design

 In this double-blinded randomized controlled clinical trial, 49 patients (20 males and 29 females, aged 40–60 years) diagnosed with peri-implant mucositis were selected from patients referred to a private periodontal office and the Faculty of Dentistry, Tabriz University of Medical Sciences. The patients were blinded to the type of treatment they received randomly (ATV or placebo gel), and the examiner was unaware of the patients’ allocation to the test or control groups. The researcher was aware of the interventions administered.

 The study protocol was initially approved by the Institutional Ethics Committee of the Islamic Azad University of Medical Sciences Tabriz Branch (protocol number IR.IAU.TABRIZ.REC.1401.198) and was conducted in compliance with the Helsinki Declaration of 1975, as revised in 2013. The study findings were reported according to the 2010 CONSORT guidelines.

 The study was officially registered with the local World Health Organization Registry Network under the code IRCT20220510054805N1. Following ethical approval, all the individuals were duly informed verbally, and written informed consent was obtained for their participation in the study.

###  Inclusion criteria

1. Age ≥ 18 years
2. Severe peri-implant mucositis or mild peri-implantitis (presence of bleeding on probing [BOP], PD > 3 mm, no soft tissue recession with or without minimal crestal bone loss ≤ 2 mm on periapical radiographs)
3. Functioning implants for ≥ 1 year^20^

###  Exclusion Criteria

1. Pregnancy and breastfeeding
2. Current and former smokers
3. Uncontrolled diabetes mellitus and immune-compromised systemic diseases
4. Presence of active periodontal disease after primary treatment
5. Use of systemic or topical antibiotics during the previous 3 months
6. Regular use of anti-inflammatory drugs in the last 3 months
7. Use of bisphosphonate, phenytoin, calcium antagonists, cyclosporine, comedo, warfarin, and heparin
8. Radiation to the head and neck region
9. History of allergy to the statin group of drugs
10. Statin therapy
11. Abuse of alcohol and drugs
12. Failing or refusing to sign an informed consent form^20^

###  Patient grouping

 Forty-nine patients were selected based on the selection criteria, and after enrollment by an examiner, the patients were randomly allocated to either the test or control group. The randomization method was simple randomization and conducted using the RandomIZE Randomization tool app. The sample size was estimated to compare the average of the two groups from the respective formula with 95% confidence and 80% power, and an effect size equivalent to that of a similar study by Pradeep et al^21^ equaled 21 participants in each group. Owing to the existence of three stages of follow-up and the possibility of dropping samples by 30%, the final sample size increased to 27 people in each group and 54 in total. Throughout all analyses of the research findings, the investigators were not part of or aware of the randomization process.

**Figure 1:**
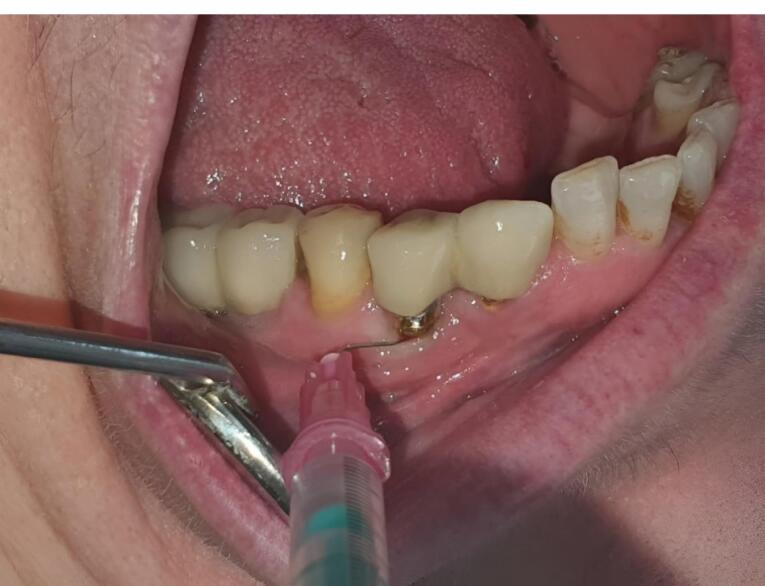
Injections of 1.2% atorvastatin or placebo gel into the pockets surrounding the implant using an insulin syringe

 After mechanical debridement in both groups, 1.2% ATV gel (1.2 mg/0.1 mL) was injected into the pockets around the implant in the test (ATV) group and placebo gel in the control (placebo) group. Mechanical debridement was performed using a plastic curette at the baseline for each patient. Prepared 1.2% ATV gel (1.2 mg/0.1 mL) or placebo gel was injected into the pockets around the implant using an insulin syringe in the test and placebo groups (Figure 1). Cyanoacrylate tissue adhesive was used to protect the area. After treatment completion, the patients were not prescribed antibiotics or anti-inflammatory drugs. They were given specific instructions for a week, including refraining from chewing hard or sticky food, brushing near the treated areas, and using any interdental aid. At one and three months after the intervention, all clinical parameters were measured again in both groups in the same area.

###  Clinical evaluation

 The evaluation involved recording various clinical parameters such as BOP, PD, CAL, and pain on probing (POP) at different time points: baseline (before mechanical debridement), 1 month, and 3 months. A custom-made acrylic stent and a color-coded periodontal probe (UNC-15, Hu-Friedy, Chicago, IL, USA) were used to ensure uniformity in measuring these parameters. An examiner blinded to each individual’s treatment recorded all the pre- and post-treatment clinical parameters.

###  Formulation of 1.2% ATV gel

**Figure 2:**
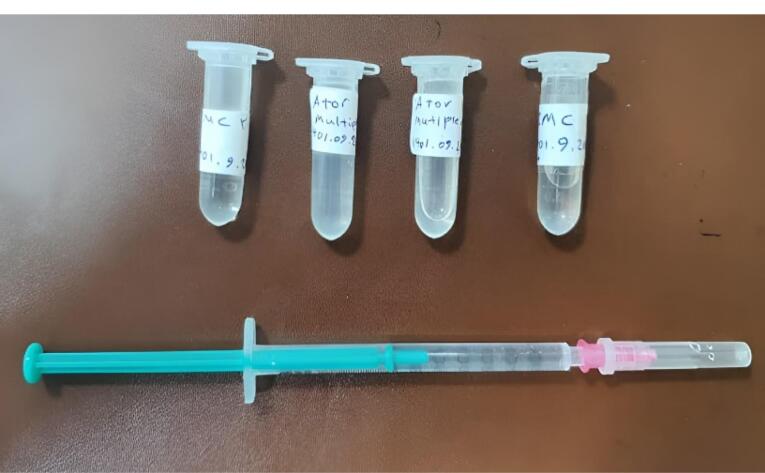
1.2% ATV gel and placebo. Due to the non-availability of atorvastatin gel in the pharmaceutical market in Iran, a pharmacist formulated the atorvastatin gel

 ATV gel was prepared by a pharmacist using standard methods described in pharmacology texts.^22-24^ After intensive in vitro investigations for optimization and stability to prepare a multiple-dose solution of isotone and sterile ATV, the gel base was first prepared with sodium carboxymethyl cellulose (2.6%) and mannitol (9%), and the pharmaceutical stock solution was prepared at a concentration of 2% in propylene glycol solvent. Subsequently, at a 4:1 ratio, the drug solution was slowly added to the gel base. If opaque, 0.5% polysorbate 80 was added to the solution to increase the solubility of the drug. Finally, 1% benzyl alcohol was added to the solution as a preservative for injectable products. All steps were performed under laminar hood and aseptic conditions. The obtained solution was stored in sterile 1.5 mL of polyethylene microtubes and was stable at 2-8 °C for up to 1 month (Figure 2).

###  Primary and secondary outcomes 

 The primary outcome of this study was the BOP. The secondary outcomes included PD, CAL, and POP.

###  Statistical analysis

 SPSS 22 was used for the data analysis. Descriptive statistics (frequency, frequency percentage, mean, and standard deviation) and inferential statistics (chi-squared test, Fisher’s exact test, and independent *t* test) were used for data analysis. An independent *t* test was used to compare the results between the two groups, and a paired *t* test was used to compare intragroup results. Statistical significance was set at *P* < 0.05.

## Results

###  Descriptive results

**Figure 3:**
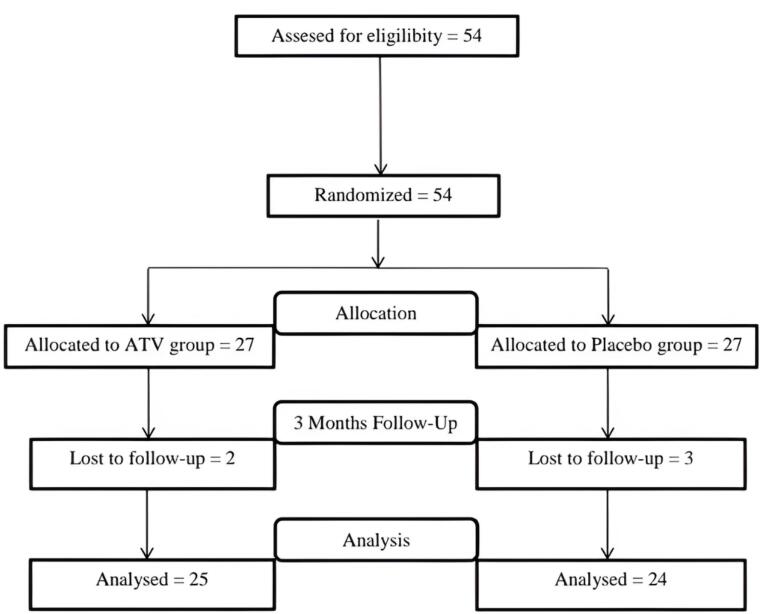
CONSORT diagram

 Forty-nine participants (one site/patient) out of 54 successfully concluded the study. Unfortunately, 5 individuals (2 in the ATV group and 3 in the placebo group) could not participate in the follow-up sessions (Figure 3). Thus, only 49 patients (20 men and 29 women) aged 41–60 years were included in the data analyses after completing the 3-month follow-up.

###  Clinical parameters

####  Inter-group results

 The clinical parameters (BOP, PD, CAL, and POP frequency distributions) at both the baseline and follow-up visits are shown in Tables 1 and 2. After one month, the independent *t* test showed no significant difference (*P* > 0.05) in the BOP index between the control and test groups. However, it significantly decreased (*P* < 0.001) in the test group after three months. There was no significant difference (*P* > 0.05) in PD between the two groups after three months, but it was significant one month after the intervention (*P* < 0.001). The CAL and POP variables showed significant differences 1 and 3 months after the intervention (*P* < 0.001).

**Table 1:** Intergroup comparisons of parameters (Mean ± SD) at baseline and 1- and 3-month follow-up visits

**Clinical parameters**	**Control group**			**Test group**		
	**Baseline**	**1 month**	**3 months**	**Baseline**	**1 month**	**3 months**
BOP (%)	76.6 ± 15.2	24.4 ± 10.2	29.4 ± 9.4	79.4 ± 13.2	28.2 ± 8.9	49.1 ± 15.4
PD (mm)	5.4 ± 0.7	3.9 ± 0.9	3.6 ± 0.8	5.4 ± 0.6	3.2 ± 0.8	3.4 ± 0.6
CAL (mm)	6.0 ± 0.7	5.5 ± 0.9	5.9 ± 0.6	6.9 ± 0.8	4.1 ± 0.6	4.6 ± 0.5
POP (cm)	5.5 ± 1.7	4.7 ± 0.7	5.6 ± 1.7	4.5 ± 1.4	3.8 ± 1.1	3.6 ± 1.0

BOP: bleeding on probing; PD: pocket depth; CAL: clinical attachment level; POP: pain on probing.

**Table 2:** Intergroup comparisons of parameters (*P* value) at baseline and follow-up visits

**Parameters**	**Baseline (** ***P*** **)**	**One-month follow-up (** ***P*** **)**	**Three-month follow-up (** ***P*** **)**
BOP	0.499	0.171	0.0001
PD	0.74	0.004	0.252
CAL	0.0001	0.0001	0.0001
POP	0.478	0.001	0.001

BOP: bleeding on probing; PD: pocket depth; CAL: clinical attachment level; POP: pain on probing.

####  Intra-group results

 A comparison of intra-group results using a paired t-test showed a significant difference in PD one and three months after the intervention (*P* < 0.001). No significant difference (*P* > 0.05) was found in CAL in the control group at baseline and one and three months after the intervention. However, within the test group, the difference was statistically significant (*P* < 0.001). A comparison of the BOP results showed a significant difference (*P* < 0.001) one and three months after the intervention. The POP results were significantly different (*P* ≤ 0.05) one month after the intervention, both within the control and test groups. There was no significant difference (*P* > 0.05) in the control group three months after the intervention, but the difference was significant (*P* < 0.05) in the test group (Table 3).

**Table 3:** Intra-group comparisons of parameters at baseline and 1- and 3-month follow-up visits

**Clinical parameters**	**Control group**		**Test group**	
	**One month from baseline**	**Three months from baseline**	**One month from baseline**	**Three months from baseline**
	**(** ***P*** **)**		**(** ***P*** **)**	
BOP (%)	0.0001	0.0001	0.0001	0.0001
PD (mm)	0.0001	0.0001	0.0001	0.0001
CAL (mm)	0.067	0.426	0.0001	0.0001
POP (cm)	0.026	0.862	0.050	0.004

BOP: bleeding on probing; PD: pocket depth; CAL: clinical attachment level; POP: pain on probing.

## Discussion

 Statins have antimicrobial activity against periodontal pathogens and exhibit anti-inflammatory and immunomodulatory effects.^25^ Animal and clinical studies have supported the idea that statins can be used as an adjunctive treatment to scaling and root planing (SRP) to manage periodontal disease, including chronic periodontitis.^18,26^ Numerous studies have investigated the effectiveness of statins on various clinical parameters of periodontitis.^27^ Due to the pleiotropic (cholesterol-independent) effects of statins, such as immunomodulatory and anti-inflammatory effects, they are expected to improve periodontal clinical outcomes.^28^ Several studies have reported positive clinical effects, such as reduced PD, CAL, and BOP, with local administration of statins. Therefore, statins are considered a valuable adjunct to non-surgical and surgical treatments for periodontal disease.^18,26,27^

 The current study evaluated the clinical efficacy of 1.2% ATV gel as a supplement to mechanical debridement in treating peri-implant mucositis. Compared with the placebo gel, the results revealed a significant improvement in clinical parameters. To the best of our knowledge, no study has directly compared the use of 1.2% ATV gel in treating peri-implant mucositis.

 In contrast, Saxlin et al^29^ investigated the dual effects of statins on the periodontium. Their study revealed that statin use was associated with a higher risk of deep periodontal pockets in individuals without BOP. However, Kumari et al^30^ discovered that using a local 1.2% ATV gel significantly improved clinical and radiographic parameters compared with a placebo gel. Similar results were also reported by Lindy et al,^31^ who found that ATV or simvastatin led to 37% fewer pathological periodontal pockets than in the control group. In addition, animal models have suggested that statins have a beneficial impact on ligature-induced alveolar bone loss.^32^ In the current study, BOP significantly decreased from baseline to three months, indicating that ATV may have anti-inflammatory properties.

 In the current study, no significant difference (*P* > 0.05) was observed in the BOP index between the control and test groups at the one-month follow-up. However, after three months, the difference was significant (*P* < 0.001) in the test group. However, the results for the PD variable one month after the intervention were significant (*P* < 0.001). There was a significant difference in CAL and POP between the control and test groups one and three months after the intervention (*P* < 0.001).

 Studies using local statins have reported significant improvements in clinical periodontal outcomes compared with those using SRP. The subgingival release of statins allows for high concentrations and low doses of drugs in the periodontal tissues, leading to high patient acceptance and the ability to control the long-term release of therapeutic agents at the target sites without causing systemic side effects.^33^ Compared with oral administration, which results in rapid absorption and entry of the drug into the circulation, local application of the drug is preferred.^34,35^ Therefore, it is safer to administer the drug locally, and clinical results have demonstrated that it improves chronic periodontitis.^27^

 Bertl et al^26^ found that the type of statin used was associated with periodontal outcomes. One study showed that rosuvastatin was the most effective, whereas another reported statistically significant effects of ATV. In two clinical trials evaluating the application of statins as an adjunct to SRP, rosuvastatin produced the best results regarding clinical and radiographic parameters such as PD reduction, CAL gain, and radiographic defect fill.^27^ The superior clinical advantages of rosuvastatin over ATV may be attributed to its stronger anti-inflammatory effect, which results in a greater reduction in C-reactive protein levels.^36,37^

 Simvastatin is the most commonly used statin in clinical trials and is administered locally at a concentration of 1.2%. Numerous studies have shown significant improvements in clinical and radiographic results when using simvastatin.^27^ Retrospective studies have also shown that patients with severe chronic periodontitis who were treated with simvastatin or ATV had lower PD indices than those who did not receive statins.^38^ In addition, a recent study by Fajardo et al^39^ indicated that ATV may reduce alveolar bone loss and tooth mobility in individuals with periodontal disease. Goes et al^40^ reported that ATV could prevent alveolar bone loss in rats with ligature-induced periodontitis.

 Pradeep et al^21^ evaluated the use of 1.2% ATV gel as a supplement to SRP for treating suprabony defects in patients with chronic periodontitis. The ATV group showed a significant reduction in clinical parameters such as BOP, PD, and CAL at the 3-, 6-, and 9-month follow-ups compared to the placebo group, indicating the anti-inflammatory effect of ATV.

 It is recommended that more samples be used in clinical studies and that longer follow-up periods and investigations of inflammatory biomarkers be conducted.

## Conclusion

 Overall, the clinical parameters of the peri-implant mucosa improved using 1.2% ATV gel as an adjunct to mechanical debridement. The results of this study support the additional application of ATV gel for the treatment of peri-implant mucositis. By injecting 1.2% ATV gel into the pockets around implants with peri-implant mucositis, this clinical trial showed that it significantly reduced BOP, PD, POP, and CAL gain when used with mechanical debridement, compared with placebo gel. This may provide a new approach for treating the inflammation caused by peri-implant mucositis. The results of this study must be confirmed in long-term, multicenter, randomized, controlled clinical trials.

## Acknowledgments

 The authors used Cloud.trinka.ai to check grammar and refine the presentation of crucial information in the introduction and discussion sections.

## Competing Interests

 The authors declare that they have no competing interests.

## Consent for Publication

 Not applicable.

## Data Availability Statement

 The data from the reported study are available upon request from the corresponding author.

## Ethical Approval

 The study was approved by the Institutional Ethical Committee of the Islamic Azad University of Medical Sciences Tabriz Branch (protocol number: IR.IAU.TABRIZ.REC.1401.198) and registered with the local World Health Organization Registry Network (IRCT20220510054805N1).
